# Acute exacerbation of OCD symptoms precipitated by media reports of COVID-19

**DOI:** 10.1017/ipm.2020.61

**Published:** 2020-05-21

**Authors:** I. French, J. Lyne

**Affiliations:** 1 Wicklow Mental Health Services, Newcastle Hospital, Greystones, Co., Wicklow, Ireland; 2 Department of Psychiatry, Royal College of Surgeons in Ireland, 123 St. Stephen’s Green, Dublin 2, Ireland

**Keywords:** Coronavirus, COVID-19, obsessive–compulsive disorder, pandemic

## Abstract

The emergence of COVID-19 has recently dominated public discourse given its serious impact on vulnerable patient groups. Advice in relation to reducing risk of contamination has justifiably been circulated widely during the COVID-19 crisis. Contamination fear is a common obsessional theme in patients with obsessive–compulsive disorder (OCD), and there is a need for increased research on how infectious disease epidemics affect patients with OCD. We present the case of a lady in her 30s with a history of well-controlled contamination OCD who presented acutely with a significant exacerbation of OCD symptoms precipitated by media reports of COVID-19. The case highlights the potential psychological impacts of infectious disease epidemics on individuals with mental illness. We also highlight some of the risks posed to such patients in response to epidemics such as the COVID-19 crisis.

## Introduction

Novel corona virus COVID-19 has dominated media coverage and public discourse in recent months (Lai *et al.*, 2020). Robust public health containment measures have been widely implemented to prevent transmission of the virus among the global population. The impact of the COVID-19 crisis on population mental health remains unclear, and it is important that the mental health burden of this pandemic on the population is evaluated.

Obsessive–compulsive disorder (OCD) is marked by the presence of recurrent obsessional thoughts or compulsive acts (World Health Organisation, 2004). Lifetime prevalence rates of OCD are estimated at approximately 2% of the population; however, sub-threshold OCD symptoms may have a much higher prevalence in the community (Ruscio *et al.*, 2010). It is recognised that there are challenges in diagnosing OCD and that this mental disorder may be underdiagnosed in the community.

OCD is associated with diminished quality of life, psychiatric co-morbidity and disability (Bobes *et al.*, 2001). The aetiological basis of OCD has yet to be fully elucidated and is likely multifactorial. Studies suggest that genetic influences account for between 27% and 47% of the variance observed in the adult OCD population, with higher estimates for childhood OCD (Browne *et al.*, 2014). Many candidate genes have been researched to date; however, none have reached significance (Pauls, 2010). Several studies have also implicated disruption to the cortico-striato-thalamo-cortical (CSTC) circuitry in the pathogenesis of the disorder (Harrison *et al.*, 2009; Fitzgerald *et al.*, 2010; Dunlop *et al.*, 2016).

Beyond the core diagnostic features of obsessions and compulsions, symptom presentation in OCD is highly heterogeneous. Factor analysis suggests that contamination obsessions and cleaning compulsions represent a discrete cluster of symptoms with longitudinal stability in the adult OCD population, and fear of contamination is the most frequently reported obsession in OCD (Murphy *et al.*, 2010). Contamination fear is often associated with compulsive rituals such as repetitive hand washing, cleaning and taking disproportionate measures to reduce exposure to perceived sources of contamination (Rasmussen & Eisen 1992). Obsessional beliefs, centred on overestimation of threat and heightened disgust sensitivity may also be implicated in contamination fear in OCD (Cisler *et al.*, 2010).

Research published to date on the mental health effects of COVID-19 has focussed on the psychological effects of public health containment measures such as social distancing, self-isolation and quarantining, as well as the mental health effects of the epidemic on frontline healthcare staff (Carvalho *et al.*, 2020; Zhou, 2020). There has been little focus on the effect of COVID-19 for patients struggling with contamination OCD who would be expected to have increased vulnerability to the psychological effects of the COVD-19 pandemic. Exacerbation of OCD symptoms has been reported following previous epidemic disease outbreaks such as SARS, MERS and influenza. There have been anecdotal reports of increasing numbers of patients presenting with relapse of OCD symptoms in the midst of these epidemics (Banerjee, 2020; Kumar & Somani, 2020). In this article, we present the case of a lady in her 30s with a history of well-controlled contamination OCD who presented acutely to a psychiatric hospital with a significant exacerbation of OCD symptoms precipitated by media reports of COVID-19.

## Case report

The patient, AB, is a lady in her 30s who presented for acute assessment to her local psychiatric hospital following referral from her General Practitioner in February 2020, prior to the reporting of any COVID-19 cases in Europe. She presented as acutely distressed and was wearing gloves and an infection control mask throughout the initial assessment. She described a 3-week history of significant deterioration of underlying but relatively well-controlled OCD symptoms precipitated by media reports of the emergence of COVID-19 variant coronavirus in China. The patient described increasing functional limitation as a result of her OCD symptoms including reluctance to leave her home for fear of contagion, intensification of ritual hand washing and excessive cleaning (consuming several hours per day), almost complete avoidance of social interactions including with friends or family, dropping out of a local educational course and eating only canned foods due to the lower perceived risk of contamination with COVID-19. These functional limitations represented a significant deterioration from baseline and had emerged over a 3-week period. Furthermore, the patient’s worsening OCD symptoms were associated with significant psychological distress including panic symptoms and passive death wish. AB exhibited good insight into the nature of her OCD symptoms and spoke of her difficulty in cognitively re-appraising her intrusive thoughts in light of the public discourse surrounding COVID-19.

AB is single, unemployed and lives alone in rental accommodation. She reported a history of child sexual abuse (CSA), significant childhood separation anxiety, emetophobia (fear of vomiting) and depressive episodes throughout her 20s. She reported that her OCD symptoms had emerged in her early 20s and had fluctuated at this time. Fear of contamination was the predominant obsession and excessive hand washing the predominant compulsion; however, preoccupation with symmetry and pathological doubt with associated checking behaviours was also present to a lesser extent. She was treated as an outpatient for OCD approximately 10 years prior to the current presentation with Sertraline 150 mg daily and Cognitive Behavioural Therapy (CBT), with significant effect. The Sertraline was subsequently self-discontinued secondary to side effects. AB had also attended counselling for trauma related to previous CSA.

AB satisfied ICD-10 criteria for a diagnosis of OCD. She did not meet ICD-10 criteria for current co-morbid depressive or other anxiety disorders. Following the initial emergency assessment, AB was commenced on Mirtazapine 15 mg nocte and was prescribed a short course of Alprazolam 250 mcg as required to treat her acute distress. At a subsequent psychiatric assessment, the mirtazapine was titrated to 30 mg, and an urgent referral for psychological assessment was made with a view to commencing CBT targeting OCD symptoms. At outpatient follow-up 2 weeks later, AB reported some initial improvement; however, news reports of the emergence of COVID-19 cases within Europe had briefly triggered a deterioration in her mental state with intensification of compulsive rituals, avoidance behaviours and psychological distress. AB described a reduction in the emotional reactivity to her obsessional thoughts, however reported that her compulsive acts continued. Augmentation with oral Risperidone was offered; however, the patient declined same, instead opting for dose titration of Mirtazapine to 45 mg nocte. Her mental state improved with this initial medication input, and she also reported feeling more assured as increased public health measures were implemented in Ireland to address the emerging COVID-19 crisis.

## Discussion

This case describes the role of an infectious disease epidemic in precipitating a significant deterioration in a patient with OCD with predominant contamination obsessions and associated compulsions. Of note, the deterioration in mental state occurred prior to the arrival of COVID-19 in Europe, suggesting that it was predominantly increased media reporting of COVID-19 which precipitated her relapse in symptoms. The case raises several important points warranting further consideration.

In response to the COVID-19 pandemic, public health authorities have implemented public health measures and sought to increase public awareness around the importance of frequent hand washing and the observation of appropriate social-distancing measures. In this context, it is important to distinguish between the rational adaptive behavioural modifications undertaken by individuals in response to the pandemic, which are vital from a public health perspective, and the obsessional, irrational thoughts and compulsive acts exhibited during relapse of OCD. The presence of significant psychological distress in response to obsessional thoughts is a key feature of OCD and may predispose this patient cohort to a variety of risks. Some of the risks identified based on the current case include increasing isolation beyond the public health need for self-isolation; avoiding certain food types or restricting food intake to avoid contamination; the emergence of co-morbid conditions such as depression, anxiety or panic symptoms and the risk of misadventure by engaging in irrational acts to counter perceived risk of contamination. Of note, figures published recently by the United States Centre for Disease Control (CDC) show a significant spike in unintentional poisonings with household sanitising agents in recent months and there have been several media reports documenting cases of physical injury and death caused by overzealous or inappropriate use of cleaning products (Chang *et al.*, 2020). Possible psychiatric causes of this remain unclear but merit consideration. A further consideration is that patients with predominant contamination obsessions might in theory actively avoid seeking medical and psychiatric attention due to a perceived heightened contamination risk in healthcare facilities, especially during periods of pandemic infectious disease.

There was also notable increase in suicidal thinking in the current case. Risk of suicidal acts may increase in response to psychological distress or feelings of entrapment. There is evidence to suggest that patients with predominant contamination obsessions and compulsive cleaning exhibit higher rates of suicidal ideation than patients presenting with other predominant obsessive compulsive themes (Chaudhary *et al.*, 2016). There are several other risk factors associated with the increased risk for suicidality in this patient cohort including female sex, co-morbid depression and personality pathology (Breet *et al.*, 2019).

To date, there is little research data on how best to manage patients with OCD who develop an exacerbation of symptoms in response to the current COVID-19 pandemic. Several factors might hinder the traditional modes of delivery of psychiatric care in the current context including the effect of staff shortages and public health containment measures as a result of COVID-19 as well as the risk that vulnerable patients will not seek necessary psychiatric care due to perceived heightened contamination in healthcare facilities. Alternative ways to deliver care, such as using telemedicine with videoconferencing facilities, should be considered (Doarn, 2018). Telephone consultations may be a reasonable alternative in the absence of videoconferencing facilities. Telemedicine approaches have the dual benefit of nullifying the potential for transmission of infectious disease while giving patients assessment and treatment where they may otherwise be unable to access such care. Moreover, psychological intervention and support may also be delivered to patients via telemedicine, including CBT (Mohr *et al.*, 2008). Given the potential for mental state deterioration in the midst of the on-going COVID-19 pandemic as demonstrated in this case, it may be necessary for physicians to identify at-risk patients who attend their practice and to take proactive measures to ensure that these patients are coping adequately from a mental health perspective.

Beyond the logistical challenges of treatment provision due to need for social distancing, traditional diagnostic and psychological formulation for OCD are complicated by the fact that COVID-19 poses real risk to individuals and to society. Therefore, the line between what constitutes an individual’s rational adaptive response to the epidemic and an irrational and maladaptive one is not clearly defined. Degree and persistence of psychological distress in response to contamination fears, as well as engagement in self-deleterious infection control measures might be examples of maladaptive responses requiring psychological intervention such as cognitive behavioural approaches.

Traditional behavioural interventions for OCD, such as exposure and response prevention which involve graduated real-world exposure to the feared stimulus (i.e. sources of contamination), may need to be substituted with approaches that encourage the client to imagine exposure to the feared stimulus. It has been suggested that the imaginal approach to exposure and response prevention provides less potent disconfirmation than that achieved by an *in vivo* approach; however, it remains an alternative approach where real-world exposure is not feasible (Gillihan *et al.*, 2012).

In the current case, antidepressant pharmacotherapy was the principal initial treatment strategy employed which resulted in gradual stabilisation of symptoms, reduction in levels of distress and improvement of the patient’s functional capacity over time. The initial stabilisation in mental state will allow improved chance for engagement with psychological therapies once COVID-19 restrictions are lifted.

## Conclusion

This case report outlines a possible clinical effect related to the onset of an infectious pandemic and the associated media reporting. Further research is needed to determine other potential mental health effects of the recent COVID-19 pandemic, and these effects should become more apparent in the coming months. In the meantime, it is important that clinicians and policymakers are aware of the potential increased risk of relapse among those with an underlying OCD diagnosis during infectious disease pandemics and that proactive efforts are made to provide necessary supports to this vulnerable population.
